# A new genetic mechanism of natural gas accumulation

**DOI:** 10.1038/s41598-018-26517-y

**Published:** 2018-05-29

**Authors:** Chengyu Yang, Zhiyong Ni, Tieguan Wang, Zhonghong Chen, Haitao Hong, Long Wen, Bing Luo, Wenzhi Wang

**Affiliations:** 1 State Key Laboratory of Petroleum Resources and Prospecting, China University of Petroleum, Beijing, 102249 China; 2 School of Geoscience, China University of Petroleum, Qingdao, Shandong 266580 China; 3 Exploration and Development Research Institute of Southwest Oil & Gasfield Company, PetroChina, Chengdu, Sichuan 610041 China

## Abstract

Natural gas of organic origin is primarily biogenic or thermogenic; however, the formation of natural gas is occasionally attributed to hydrothermal activity. The Precambrian dolomite reservoir of the Anyue gas field is divided into three stages. Dolomite-quartz veins were precipitated after two earlier stages of dolomite deposition. Fluid inclusions in the dolomite and quartz are divided into pure methane (P-type), methane-bearing (M-type), aqueous (W-type), and solid bitumen-bearing (S-type) inclusions. The W-type inclusions within the quartz and buried dolomite homogenized between 107 °C and 223 °C. Furthermore, the trapping temperatures and pressures of the fluid (249 °C to 319 °C and 1619 bar to 2300 bar, respectively) are obtained from the intersections of the isochores of the P-type and the coeval W-type inclusions in the quartz. However, the burial history of the reservoir indicates that the maximum burial temperature did not exceed 230 °C. Thus, the generation of the natural gas was not caused solely by the burial of the dolomite reservoir. The results are also supported by the presence of paragenetic pyrobitumen and MVT lead-zinc ore. A coupled system of occasional invasion by hydrothermal fluids and burial of the reservoir may represent a new genetic model for natural gas accumulation in this gas field.

## Introduction

The Anyue gas field lies in the center of the Sichuan Basin in southwestern China and is one of the oldest and largest gas fields in China^1–3^. The Anyue gas field was formed by the in-situ pyrolysis of an ancient oilfield^4,5^. The petroleum within the ancient oilfield was generated from a black shale and was captured by the Sinian dolomite^2,6^. However, the natural gas charging age is still controversial^2–4,6^. The fluid inclusion method provides an effective means of determining natural gas charging ages^7,8^. Studies of hydrocarbon-bearing fluid inclusions have been carried out by many geologists and employ the bulk compositions, phase envelopes, and isochores of coeval aqueous fluid inclusions^9–14^. In this study, a group of methane fluid inclusions and the associated aqueous fluid inclusions are examined using laser Raman spectroscopy. The trapping temperatures and pressures of inclusions are determined using the intersections of isochores. Furthermore, inclusion petrography, the trapping temperatures and pressures of pure methane inclusions and the burial history of well GS6 indicate a new mode of gas generation that couples the effects of burial and hydrothermal fluid systems.

## Geological setting

**Figure 1:**
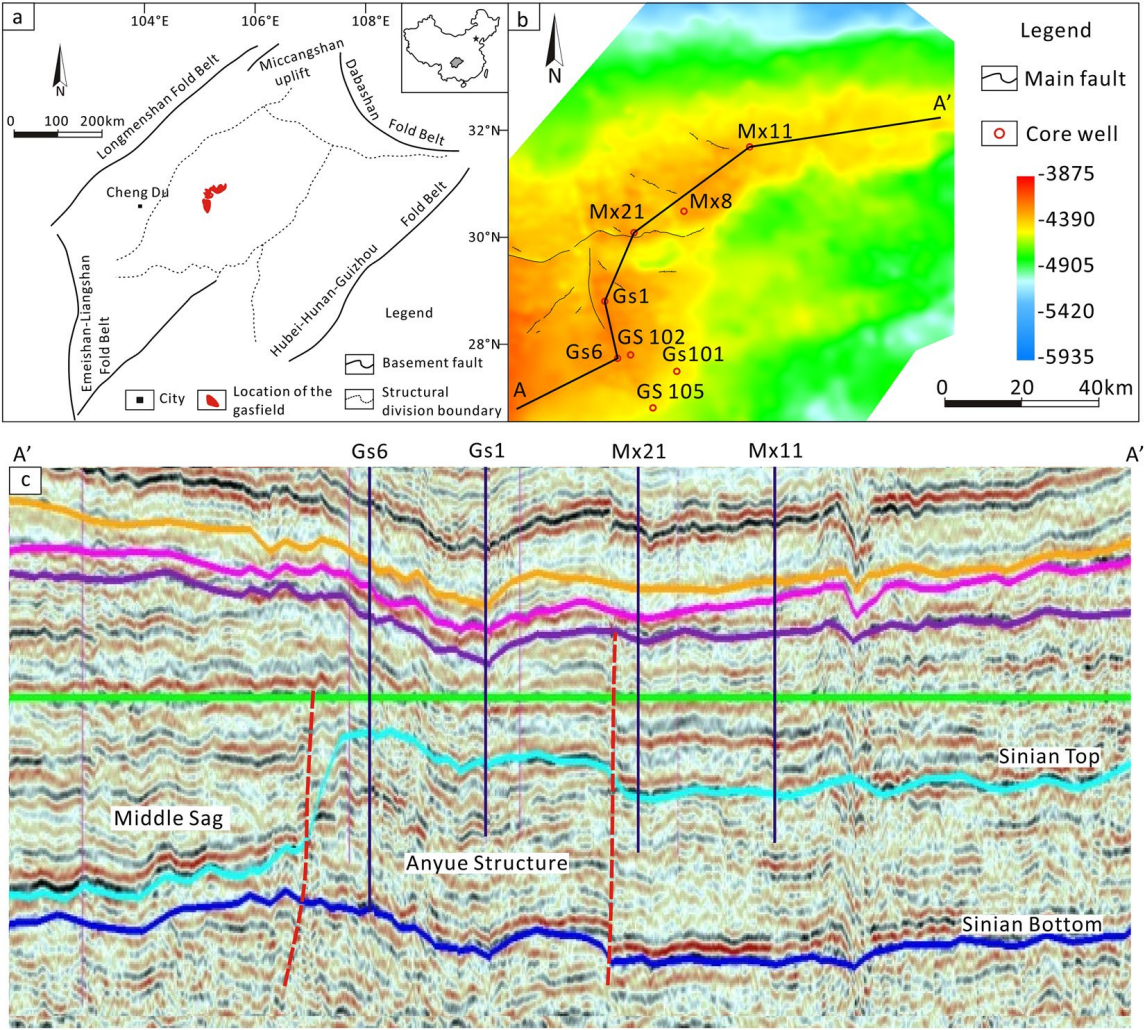
Regional setting and seismic profile of the study area.

**Figure 2:**
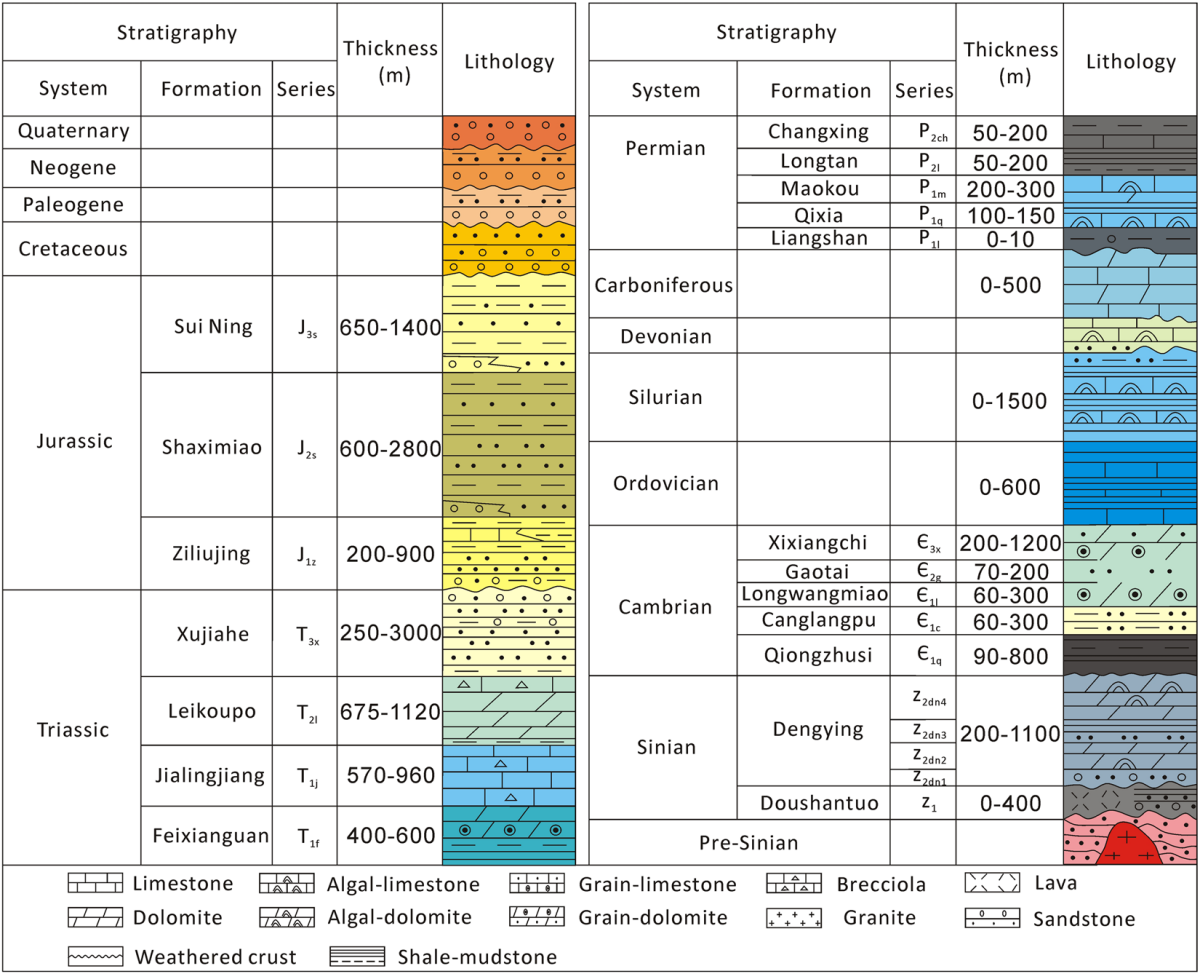
Geological column of the Sichuan Basin.

The Sichuan Basin is located in Southwestern China and has an area of approximately 260,000 km^2^ (Fig. 1). The basin is a part of the Yangzi platform, and its basement formed around 800 Ma^15,16^. A thick succession of marine carbonates overlies the basement (Fig. 2). At the same time, the Leshan-Longnvsi paleo-uplift formed in the middle of the basin and became a potential petroleum system^17,18^. The sedimentary cover of the Leshan-Longnvsi paleo-uplift includes Sinian-Ordovician marine carbonates, Permian-Triassic carbonate-clastic rocks and Triassic-Quaternary clastic rocks (Fig. 2). Marine conditions persisted on the uplift from the Sinian to the Middle Triassic (Fig. 2). Due to the lifting in Paleozoic, the Devonian to Carboniferous successions are absent on the uplift (Fig. 2). However, after the tectonic transform in the Middle Triassic, the continental succession had become the main sediment until now (Fig. 2).

The Anyue gas field is located on a paleo-high point of the Sichuan Basin^19^ (Fig. 1a,b). The thick black shale of the Qiong Zhusi Formation was deposited within the depression between two high points and became the main resource rock for two gas fields (Fig. 1c). As the shale entered the oil generation window, the oil migrated into the traps within the high points^2^. The Sinian succession was then buried to a depth of 5015–5396 m. During the burial process, the oil may have pyrolyzed into pyrobitumen and gas, which are the most common materials filling the pore space in the reservoir^2,3^. The geochemistry evidences of pyrobitumen indicate that the gas field was generated by an in-situ burial pyrolysis of paleo-oil^20,21^. The Sinian reservoir is located at the top of the Dengying Formation (Z_2dn4_) (Fig. 2). Our samples were obtained from exploration wells within the gas field, and the core samples were collected from the Z_2dn4_ Formation. The Z_2dn4_ reservoir is composed primarily of algal bindstone and mud-sized dolostone that formed as a carbonate reef along the edge of the platform^2^ (Fig. 1c). The dolostone contains a number of dissolution pores, vugs, and cracks, due to strong karstification by meteoric waters^2,3^. A moderately sulfur-rich natural gas that is primarily composed of methane is contained within these reservoir spaces^3^.

## Methods

Samples were obtain from well GS6 to determine the homogenization temperatures of fluid inclusions, and these samples originated at depths ranging from 5034.5 to 5049.6 m. The homogenization temperatures were measured using various minerals. Thirteen single-phase pure methane inclusions and 8 coeval aqueous fluid inclusions in the same authigenic quartz were examined to obtain the trapping temperatures and pressures. First, samples were cut into small pieces. These pieces were doubly polished into thin sections (<0.30 mm thick). The inclusion temperature measurements were performed using a Linkam THMSG600 heating-freezing stage following standard procedures in the State Key Laboratory of Petroleum Resources and Prospecting, China University of Petroleum, Beijing. The homogenization temperatures of both gas inclusions and the coeval aqueous fluid inclusions were measured; the freezing points of aqueous fluid inclusions in authigenic quartz were also measured. The heating rate was 15 °C/min during the initial stages of each heating run and was reduced to 0.3–1 °C/min close to the phase transitions. The salinities of the aqueous fluid inclusions were calculated using ice points of H_2_O-NaCl^22^.

## Results

### Petrography

**Figure 3:**
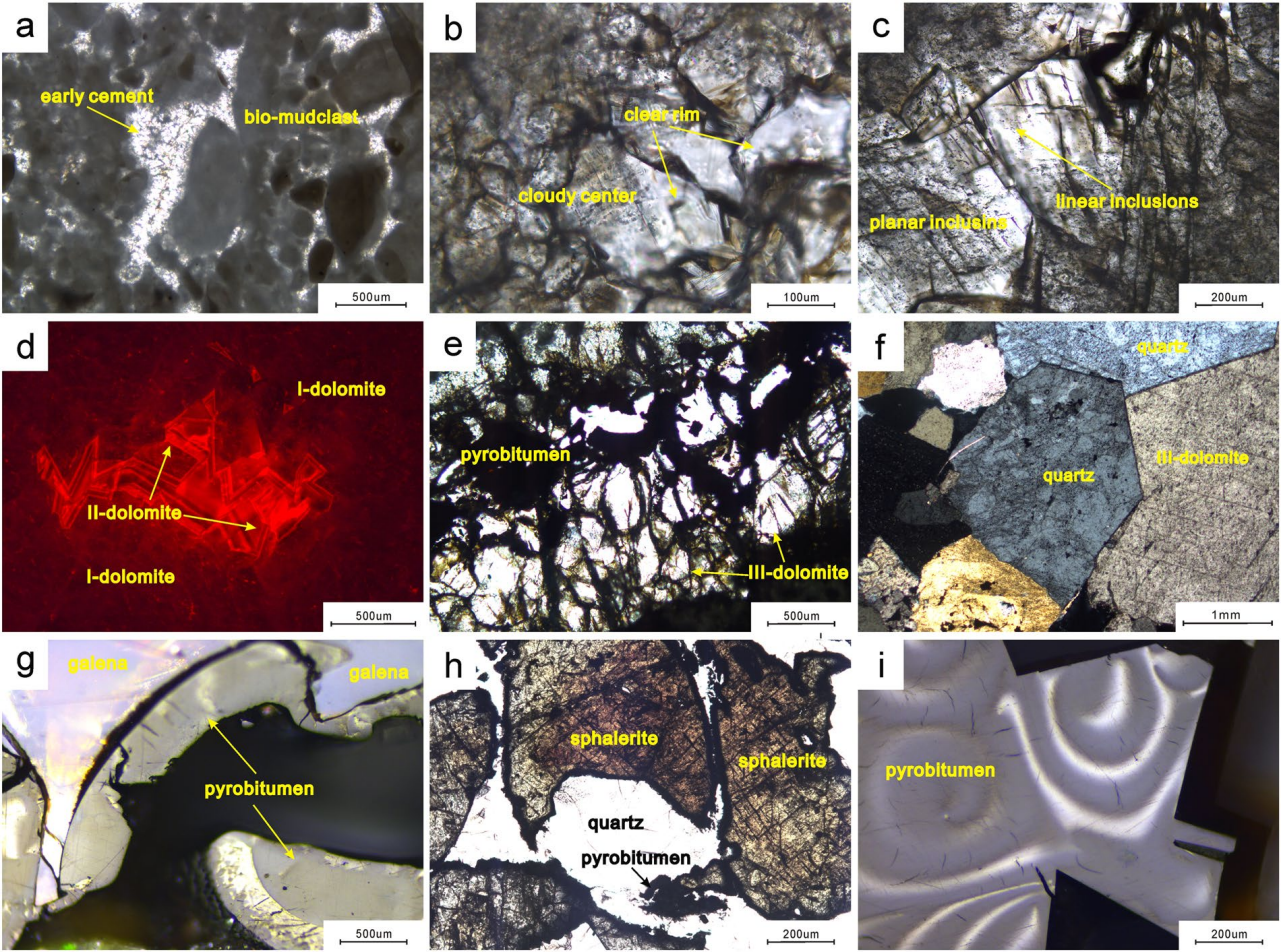
Diagenetic characteristics of the Z2dn4 Formation. (**a**) Bioclasts, mudclasts and early cement, plane-polarized light (non-polarized transmitted light), well GS6, 5048.97 m. (**b**) II-dolomite with a cloudy center and a clear rim (non-polarized transmitted light), well GS6, 5035.84 m. (**c**) Planar distribution of inclusions in a cloudy center (non-polarized transmitted light), well GS6, 5035.02 m. (**d**) Different kinds of dolomite within a pore (cathodoluminescence), well GS6, 5035.84 m. (**e**) Pyrobitumen accompanied by III-dolomite (non-polarized transmitted light), well GS6, 5049.1 m. (**f**) III-dolomite and quartz (cross-polarized transmitted light), well GS103, 5177.67 m. (**g**) Pyrobitumen associated with galena, well GS102 (non-polarized reflecting light), 5043.2 m. (**h**) Sphalerite and quartz in a lead-zinc vein (non-polarized reflecting light), well GS101, 5177 m. (**i**) Pyrobitumen with strong anisotropy (non-polarized reflecting light), well GS105, 5220 m.

Algal bindstone and mud-sized dolostone is the main rock type in the reservoir (Fig. 2). Bioclasts and mudclasts are the most common types of grains within the samples (Fig. 3a). Both the grains and the cement are composed of dolomite, and the overall rock unit is divided into three stages. The initially deposited dolomite (I-dolomite) contains tiny mudclasts and bioclasts. Although some of the mudclasts and bioclasts have been recrystallized (Fig. 3a), the I-dolomite reflects surface replacement dolomitization that changed only the composition of the carbonates^23^. The dolomite resulting from the second stage (II-dolomite) is represented by overgrowths along the pores and cracks among the mudclasts and bioclasts (Fig. 3b,c). The II-dolomite commonly grows on the sides of pores and displays an internal zonation structure in which the dolomite can be divided into two parts, a cloudy center and a clear rim (Fig. 3b–d). This type of dolomite grew during the burial stage, and the internal zonation structure reflects changes in crystal formation^23,24^. The dolomite resulting from the third stage (III-dolomite) occupies the pore centers and cracks (Fig. 3d–f). The III-dolomite is marked by very large crystals with curved crystal faces and relatively dim luminosity; it shows no internal structure under cathodoluminescence (Fig. 3d). Furthermore, the III-dolomite may be related to hydrothermal activity^25,26^. Pyrobitumen and quartz are also observed within the pores and cracks (Fig. 3e,f). Quartz is always associated with the III-dolomite and is sporadically mixed with pyrobitumen (Fig. 3f). The pyrobitumen may be related to hydrothermal activity; it coats the II-dolomite and is coeval with the III-dolomite and the quartz (Fig. 3e,f). Furthermore, the pyrobitumen is always associated with MVT lead-zinc ore^22^ (Fig. 3g,h) and shows strong anisotropy (Fig. 3i). Moreover, the pyrobitumen is anisotropic, which indicates that it formed by the^25^ coking of liquid oil^27–29^.

### Diagenesis

**Figure 4:**
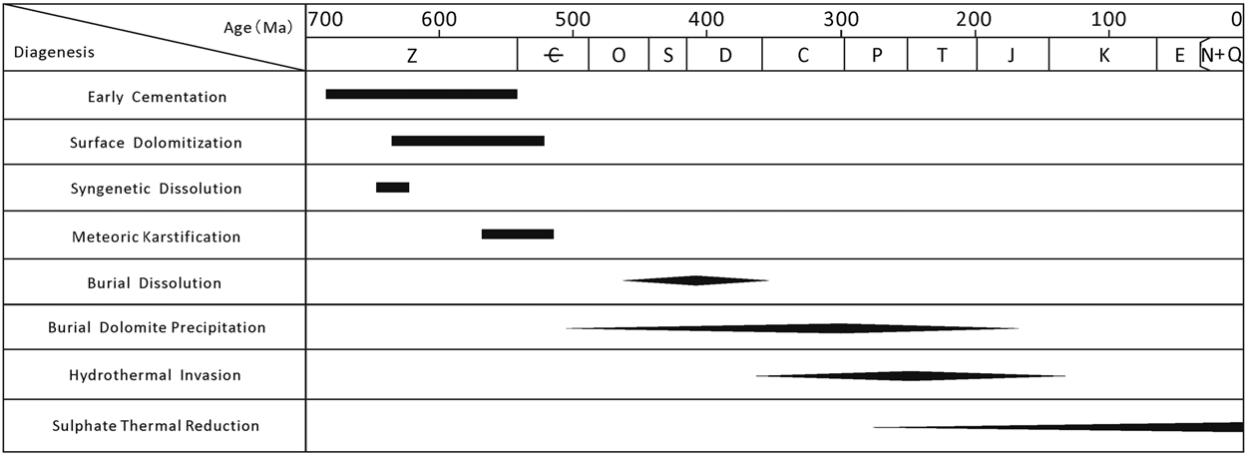
Diagenetic evolution of the Sinian carbonate reservoir rocks penetrated by well GS6 in the Anyue gas field.

Properly understanding the diagenesis of the dolomite is critical for the measurement of fluid inclusions. A clear sequence of host minerals must be identified to accurately identify the different inclusion stages^7^. The primary minerals hosted within the inclusions are dolomite and quartz. The reservoir underwent early cementation, surface dolomitization, syngenetic dissolution, meteoric karstification, burial dissolution, precipitation of dolomite during burial, hydrothermal invasion, and sulfate thermal reduction^30–33^ (Fig. 4). Furthermore, the most important stages of diagenesis for inclusion measurement are the later stages of dolomite precipitation (II-dolomite and III-dolomite). The few inclusions formed by early surface cementation, dolomitization, dolomite precipitation and dissolution could not be observed under a microscope, due to the low transparency of the minerals (Fig. 3a,b). Furthermore, the inclusions formed by early diagenesis are not related to hydrocarbon activity. The burial conditions influenced the formation of the II-dolomite, the III-dolomite and the quartz; both gas inclusions and aqueous fluid inclusions are abundant and can be observed in these phases.

### Observations of fluid inclusions

The inclusions within the dolomite reservoir are identified by their compositions. Microscopy and laser Raman spectroscopy indicate the existence of four types of inclusions: pure methane (P-type), methane-bearing (M-type), aqueous (W-type), and solid-bearing (S-type) (Fig. 5).

1. Pure methane inclusions: The P-type fluid inclusions are pure gas inclusions that contain only methane vapor. These inclusions are polygonal or ovoid in shape and range from 4 µm to 18 µm in size (Fig. 5a–c). These inclusions are commonly homogeneous and dark under plane-polarized light, although some P-type fluid inclusions are bright (Fig. 5a–c). Laser Raman spectroscopy of the P-type fluid inclusions shows a prominent peak at 2911.5 cm^−1^, which is the characteristic peak of methane (Fig. 6a). Few P-type fluid inclusions are visible in the clear rims of II-dolomite, and the majority of the P-type fluid inclusions occur in the III-dolomite and quartz, which are hydrothermal minerals. Furthermore, the P-type fluid inclusions are linearly distributed and accompanied by M-type and S-type inclusions.

**Figure 5:**
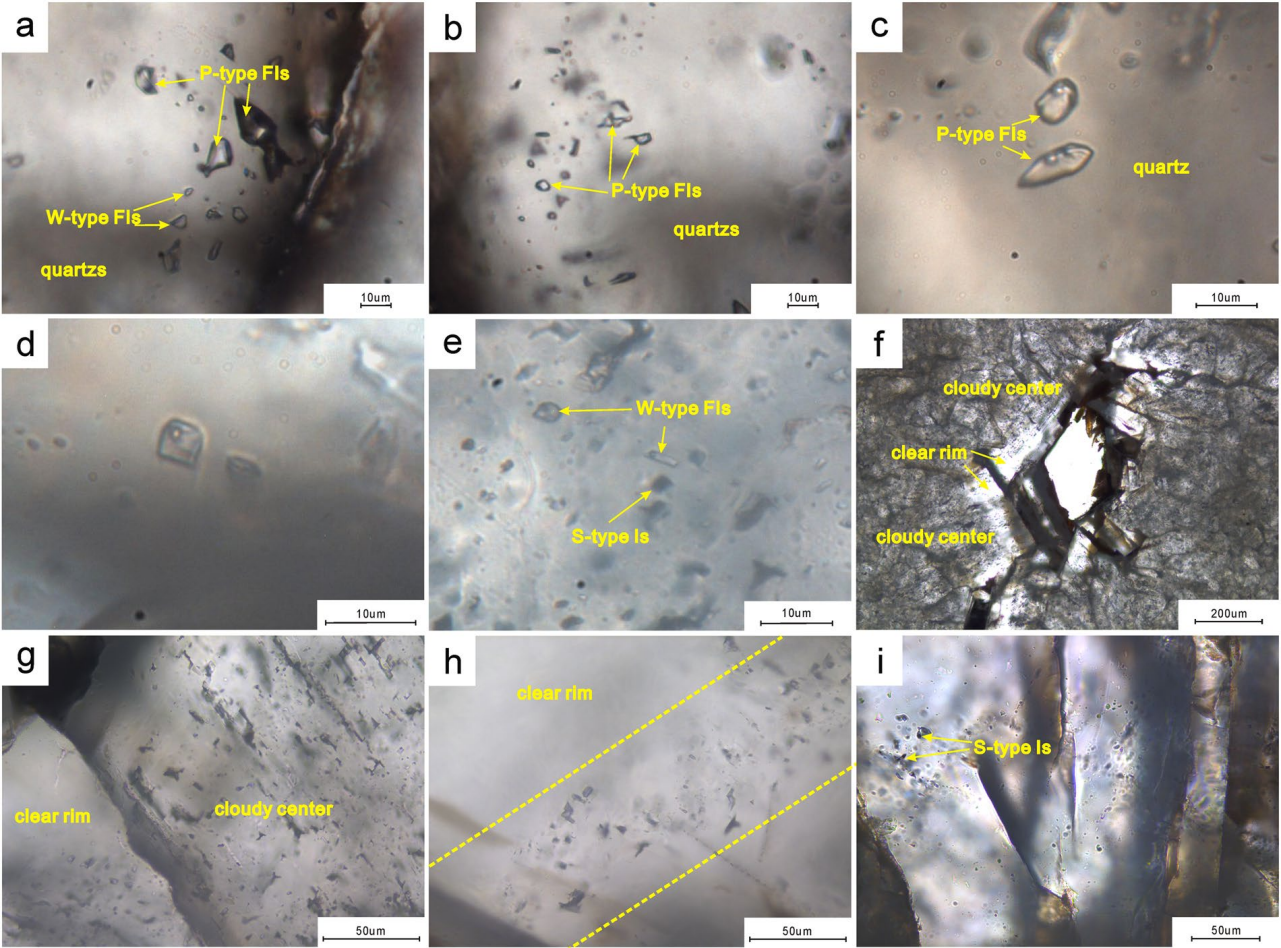
Characteristics of inclusions found in the Z2dn4 Formation of well GS6. (**a**) W-type fluid inclusions (FIs) and P-type FIs in quartz (PPL), 5049.1 m. (**b**) P-type FIs in quartz (PPL), 5049.1 m. (**c**) P-type FIs in quartz showing two-phases of liquid and vapor on freezing table, at the temperature of −115 °C (PPL), 5049.1 m. (**d**) C-type fluid inclusions within the III-dolomite, 5048.97 m. (**e**) W-type FIs and S-type inclusions (Is) in III-dolomite (PPL), 5035.31 m. (**f**) II-dolomite (PPL), 5035.31 m; g. inclusions distributed at the boundary between a cloudy center and a clear rim in II-dolomite (PPL), 5035.02 m. (**h**) linear inclusions in a clear rim of II-dolomite, plane-polarized light (PPL), 5034.48 m. (**i**) S-type Is within a clear rim of II-dolomite (PPL), 5049.1 m.
2. Methane-bearing inclusions: The M-type fluid inclusions are composed of methane gas and liquid H_2_O; the vapor phase occupies 10–30% of the total volumes of these inclusions (Fig. 5d). These inclusions show polygonal or ovoid shapes and are 4 µm to 10 µm across (Fig. 5d). Laser Raman spectroscopy shows the characteristic peak of methane in vapor bubbles within M-type fluid inclusions (Fig. 6b). The M-type fluid inclusions are commonly accompanied by P-type fluid inclusions and are present in the II-dolomite, the III-dolomite, and the quartz. Compared with the two-phase W-type fluid inclusions, the number of two-phase M-type fluid inclusions is limited.
3. Aqueous fluid inclusions: The W-type fluid inclusions are the most commonly observed in this study (Fig. 5a,e). The W-type inclusions contain gas and liquid, and the gaseous phase occupies 5–20% of the total volume (Fig. 5e). Most of these inclusions are polygonal to ovoid in shape and range from 4 µm to 15 µm in width (Fig. 5e). The W-type fluid inclusions are abundant along the boundaries of the cloudy centers and clear rims of II-dolomite, and these inclusions usually display a planar distribution under a microscope (Fig. 5f,g). Nevertheless, other aqueous fluid inclusions are linearly distributed within the clear rims of the II-dolomite and the quartz (Fig. 5h) or are found only along the cleavage planes of the III-dolomite (Fig. 5e). In addition, the W-type fluid inclusions are the most easily measurable inclusions in the samples.
4. Solid-bearing inclusions: The S-type inclusions are present within the II-dolomite, the III-dolomite, and the quartz (Fig. 5e,i). The S-type inclusions are divided into triple-phase S-type inclusions and single-phase S-type inclusions. The triple-phase S-type inclusions contain methane gas, liquid H_2_O, and pyrobitumen, are polygonal or ovoid in shape, and range from 4 µm to 15 µm in size (Fig. 6c). The single-phase S-type inclusions were observed almost exclusively in the III-dolomite. The triple-phase S-type inclusions were observed primarily in the II-dolomite. Laser Raman spectroscopy displays the characteristic peaks of pyrobitumen and methane within the S-type inclusions (Fig. 6c). The S-type inclusions are rare in the II-dolomite, and the majority of S-type fluid inclusions occur in the III-dolomite and the quartz.

**Figure 6:**
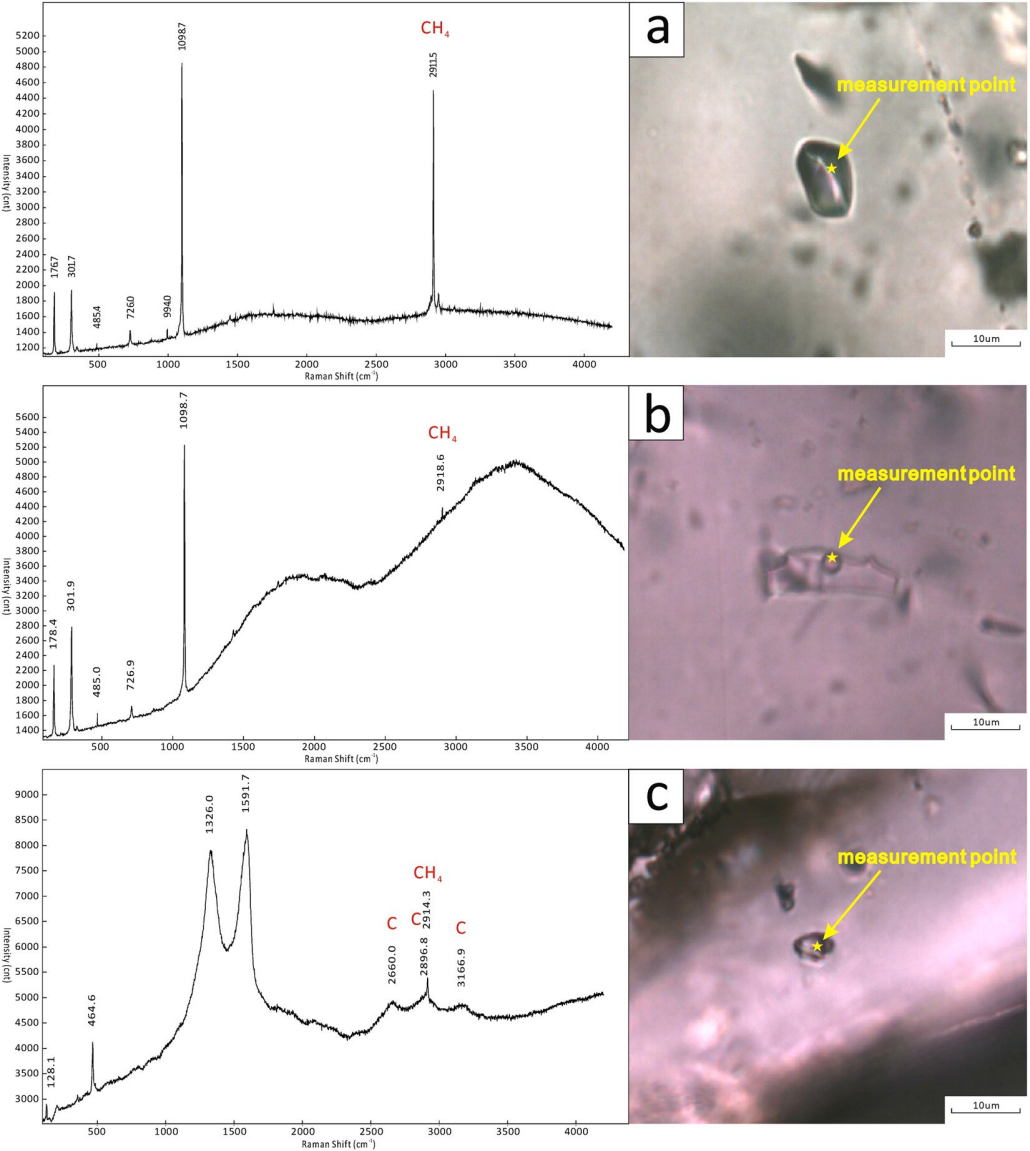
Raman spectroscopy of different kinds of inclusions in the Z2dn4 Formation of well GS6. (**a**) P-type FIs (PPL), 5049.1 m. (**b**) M-type FIs (PPL), 5049.1 m. (**c**) triple-phase S-type Is (PPL), 5049.1 m.

### Fluid inclusion microthermometry

**Figure 7:**
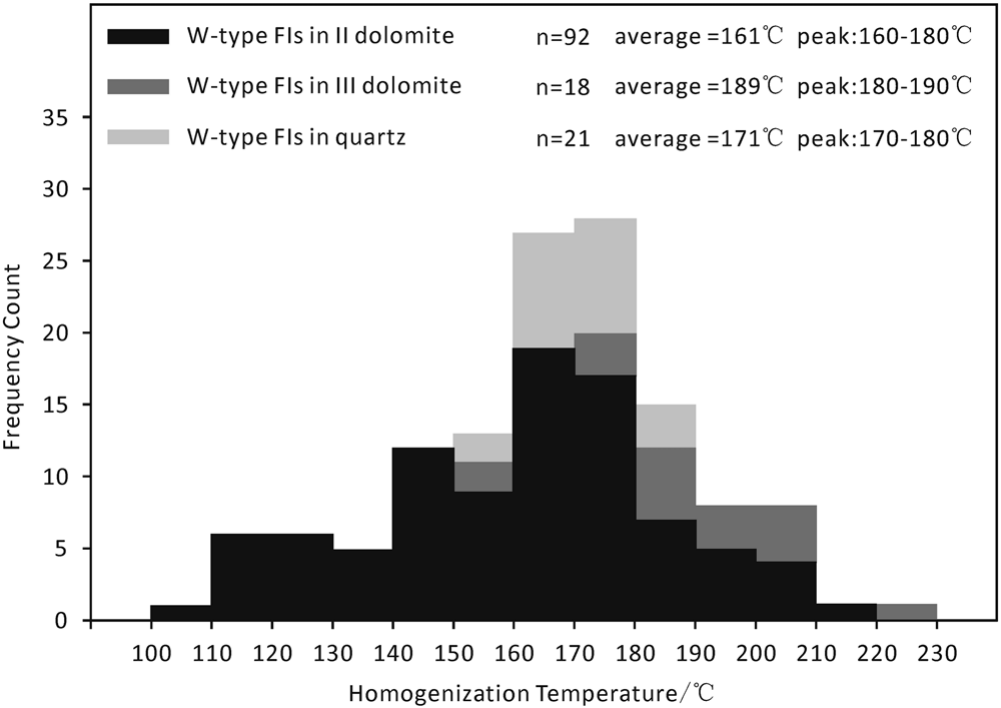
Distribution of homogenization temperatures of the W-type fluid inclusions (FIs) from Sinian carbonate reservoir samples obtained from well GS6.

Based on the sequence of the host minerals, the W-type fluid inclusions are divided into 3 groups: the W-type fluid inclusions that were observed in the II-dolomite, the W-type fluid inclusions that were observed in the III-dolomite, and the W-type fluid inclusions that were observed in the quartz (Fig. 5). Homogenization temperatures were measured for 131 W-type fluid inclusions found in the II-dolomite, the III-dolomite, and the quartz and 13 P-type fluid inclusions. The homogenization temperatures of the W-type fluid inclusions from II-dolomite range from 107 to 212 °C. The homogenization temperatures of the W-type fluid inclusions from the III-dolomite range from 158 to 223 °C, whereas the homogenization temperatures of the W-type fluid inclusions from the quartz range from 158 to 187 °C. Different groups of aqueous inclusions show different distributions of homogenization temperatures (Fig. 7). In general, the range of homogenization temperatures of the W-type fluid inclusions gradually decreases with the measured sequence from the II-dolomite to the quartz. Furthermore, the diagram shows that the W-type fluid inclusions of all of the hosting minerals homogenized at the same peak temperature from 160 to 180 °C (Fig. 7). For the P-type fluid inclusions, the samples must be cooled to measure the homogenization temperature of P-type fluid inclusions, due to the low critical point of CH_4_ (−82.6 °C). The contents of the P-type fluid inclusions changed from a single phase to two phases, and bubbles formed when the temperature was decreased below −110 °C (Fig. 5c). The contents of the P-type inclusions were homogenized to a liquid phase at temperatures varying from −82.6 °C to −105.6 °C (Table 1). The densities of the P-type fluid inclusions were calculated from the homogenization temperatures based on the work of previous studies^34,35^. These densities range from 0.173 g/cm^3^ to 0.317 g/cm^3^ (Table 1) and are greater than the critical density of methane (0.162 g/cm^3^). It is almost impossible to measure the ice point temperatures for most of the W-type fluid inclusions, due to their small sizes (<10 μm). Only 8 sufficiently large W-type fluid inclusions were selected for ice point temperature measurements (Table 1).

**Table 1:** Microthermometric data of the P-type fluid inclusions and the coeval W-type fluid inclusions noted in well GS6.

Methane FIs			Aqueous FIs			
Th (°C)	Salinity (wt% NaCl)	Density (g/cm^3^)	Th (°C)	Salinity (wt% NaCl)	Size (μ)	V_vap._ (%)
−82.6	—	0.173	187	18.5	13	15
−82.7	—	0.184	159	22.4	11	5
−82.8	—	0.189	158	18.1	13	15
−83	—	0.197	175	21.4	12	10
−82.9	—	0.194	179	23.3	12	10
−83.2	—	0.202	161	18.8	10	15
−98.5	—	0.296	173	19	11	10
−103.2	—	0.311	177	18.7	11	5
−103.7	—	0.313				
−103.9	—	0.313				
−104.2	—	0.314				
−104.4	—	0.314				
−105.6	—	0.317				

### Trapping temperatures and pressures of the P-type fluid inclusions

**Figure 8:**
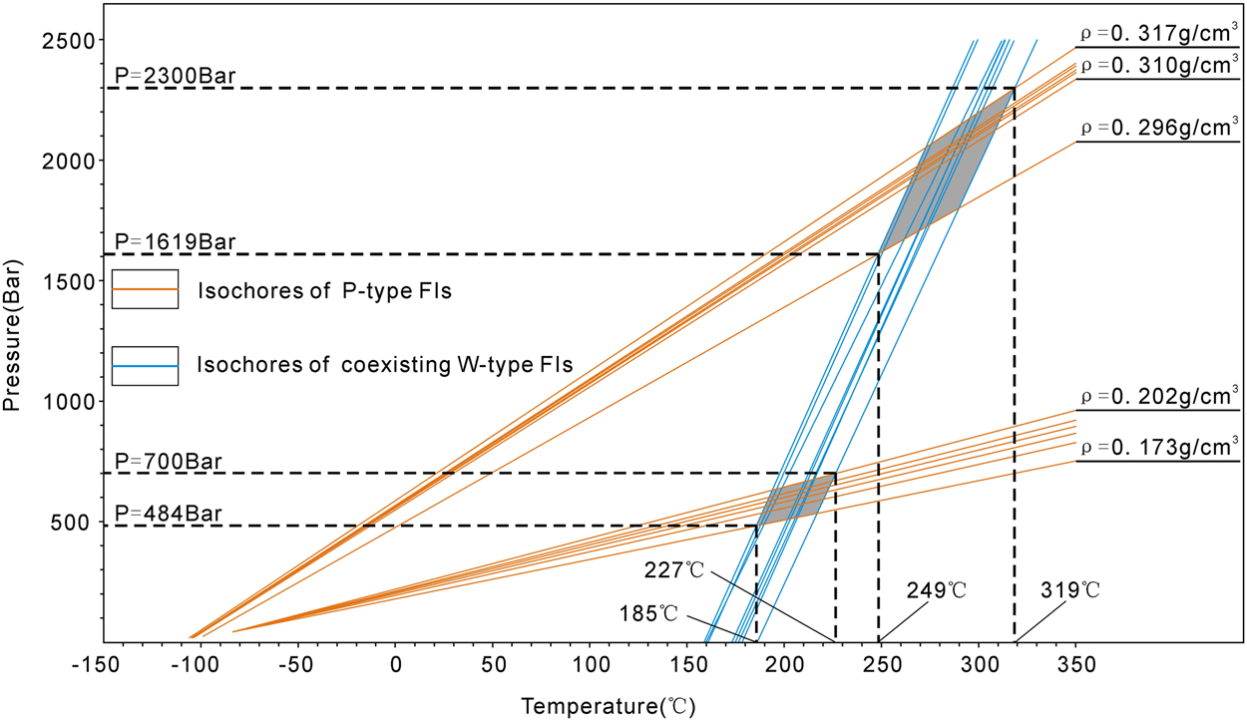
Representative isochores for P-type fluid inclusions (FIs) and isochores for coexisting W-type fluid inclusions (FIs).

The homogenization temperatures and pressures are the lowest trapping temperatures and pressures of the fluid inclusions. Because the volumes of the fluid inclusions do not vary, the temperatures and pressures of the fluid inclusions vary along isochores^36^. Furthermore, the homogenization temperatures of the P-type fluid inclusions were measured, and the salinities of the coeval W-type fluid inclusions were calculated via their freezing points^22^. Thus, the isochores of the P-type fluid inclusions and the coeval W-type fluid inclusions are drawn on a P-T graph (Fig. 8). The intersections of the two groups of isochores mark the trapping temperatures and pressures of individual pairs of fluid inclusions. Consequently, the trapping temperatures and pressures are distributed over two pairs of ranges, one of which is 185 to 227 °C and 484 to 700 bar, whereas the other is 249 to 319 °C and 1619 to 2300 bar.

### Burial history

The Leshan-Longnvsi paleo-uplift records a complex burial history^37–40^. The temperature evolution of well GS6 was reconstructed in this study using PetroMod 1D modeling software. Furthermore, 24 core samples were collected from well GS6 for Vitrinite-like reflectance measurements and were used to verify the heat flow history. Due to the absence of true Vitrinite derived from higher plants and the high maturity of the organic matter in the Sinian sedimentary rocks, reflectance values determined from vitrinite-like macerals (VLMR_o_) were used as an index of thermal maturity^41–43^. Two linear regression equations proposed by Xiao *et al*.^43^ were used to calculate Ro from the VLMR_o_ values:

1 $${\rm{Ro}}=0.28\times {\rm{VLMRo}}+1.03\,({\rm{for}}\,{\rm{VLMRo}}=0.75\mbox{--}1.5 \% )$$

2 $${\rm{Ro}}=0.81\times {\rm{VLMRo}}+0.18\,({\rm{for}}\,{\rm{VLMRo}} > 1.5 \% ).$$

**Figure 9:**
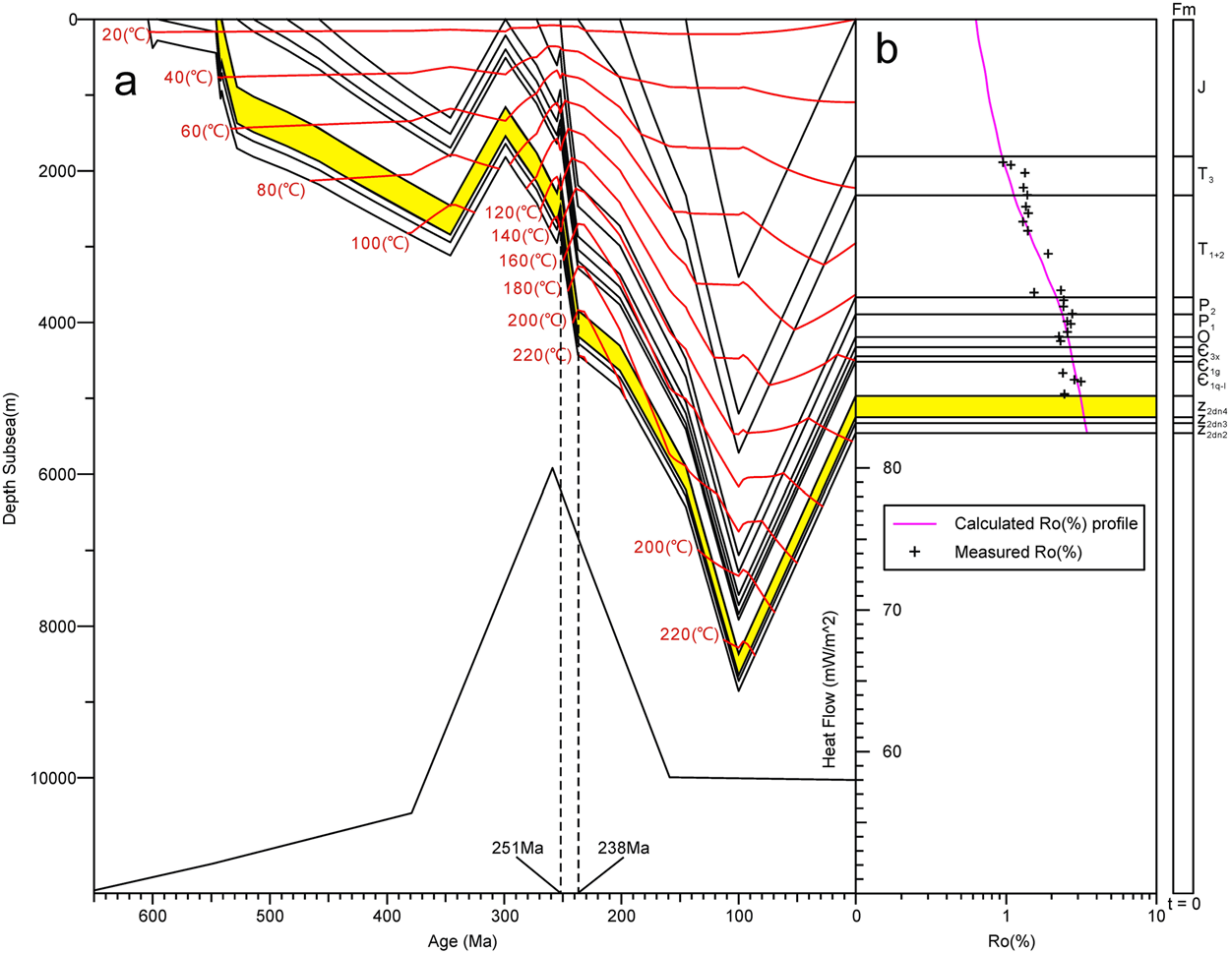
(**a**) Reconstructed stratigraphic burial, thermal histories and paleo-heat flux history plots for well GS6^47^ (**b**) (%Ro) modeled-maturity profiles.

The core samples from well GS6 revealed 5454 m of strata, which include the following formations: Z_2_dn_2_ (132 m), Z_2_dn_3_ (76 m), Z_2_dn_4_ (281 m), Є_1q-l_ (451 m), Є_1g_ (74 m), Є_3x_ (119 m), O (134 m), P_1_ (300 m), P_2_ (224 m), T_1+2_ (1346 m), T_3_ (514 m), and 1803 m of Jurassic strata^43^ (Fig. 9a). The major stratigraphic unconformity in this sequence is the Mesozoic-Cenozoic boundary. We estimate that 3400 m of Mesozoic-Cenozoic rocks are erosed, based on regional geologic and seismic data^44,45^. The Permian-Ordovician boundary is another important stratigraphic unconformity. We estimate that 1300 m of Permian-Ordovician formations are missing, according to He *et al*.^46^. There are also many minor unconformities related to other episodes of uplift and erosion. The missing sections of underlying rocks at the Z_2_dn_3_/Z_2_dn_2_ (100 m)_,_ Є/Z_2_dn_2_ (100 m), and T/P (200 m) boundaries were estimated according to the regional geology^2–4,6^. Zhu *et al*. (2015) studied the heat flow of the Sichuan basin using apatite fission track data and (U-Th)/He thermochronology^47^. The results suggest that the following heat flux history for the Sichuan Basin: (1) the heat flux was 55 ± 5 mW/m^2^ before and during the Late Carboniferous; (2) an abrupt rise in the heat flux to 80 ± 5 mW/m^2^ occurred in the Permian; (3) the heat flux gradually decreased after the Triassic, and the present day heat flux was calculated to be as low as 50 mW/m^2 ^^47^. To ensure the reliability of the modeling results, an interactive optimization process was carried out until the vitrinite-like reflectance values were matched (Fig. 9b). A good fit to the maturity data suggests that our calculated burial depths are relatively reliable.

## Discussion

### Evolution of the fluid

As mentioned above, inclusions are observed in different host minerals, which show a clear precipitation sequence. Thus, the relationship of the P-type, M-type, and W-type fluid inclusions and the S-type inclusions in the different host minerals indicate the evolution of the fluid. Large amounts of solid bitumen (pyrobitumen) were observed in the hydrothermal mineral veins (Fig. 3f–h), and a majority of the P-type fluid inclusions and the S-type inclusions are present in the hydrothermal dolomite-quartz veins, suggesting that the hydrocarbon evolution included the cracking of paleo-oil and natural gas related to the epizonogenic hydrothermal fluid^48,49^. The absence of petroleum inclusions results from the high heating temperatures, which are supported by the elevated trapping temperatures of the P-type fluid inclusions (~319 °C). While the II-dolomite was precipitating, the liquid oil may have been captured. The oil inclusions then pyrolyzed at high temperatures and formed the triple-phase S-type inclusions in the II-dolomite. Furthermore, the homogenization temperatures of the fluid inclusions within the quartz are within 10–15 °C of one another, and the salinity values fall within a range of 6 wt% NaCl. Therefore, the distribution of homogenization temperatures and the salinities of different inclusions provide the best evidence of the original temperature conditions^7^.

The trapping temperatures and pressures were obtained from the intersections of the isochores of the P-type and the coeval W-type fluid inclusions in the quartz (Fig. 8). The two ranges of trapping temperatures of the natural gas are 249–319 °C and 185–227 °C. The later stage trapping temperature (319 °C) is higher than the highest burial temperature (230 °C). We infer that the highest trapping temperature represents the invasion of deep epizonogenic hydrothermal fluids^48,49^. The earlier stage trapping temperature (185 °C) is accompanied by lower trapping pressures. We infer that the lowest trapping temperature represents a fluid system that was equilibrated between deep epizonogenic hydrothermal fluids and formation water (Fig. 9a). Because the formation temperature was lower than the hydrothermal fluid temperature, the temperature of the hydrothermal fluids decreased, and these fluids equilibrated with the formation water. During this process, the quartz grew continuously and trapped the low-temperature P-type fluid inclusions. The equilibrating process is indicated by the methane inclusions with different trapping temperatures (Fig. 8). The trapping pressures in the late and early stages of the P-type inclusions range from 1619 to 2300 bar and 484 to 700 bar, respectively (Fig. 8). The shift in pressure can be interpreted as reflecting alternating lithostatic-hydraulic fluid systems^50^. The lowest trapping temperature exceeds the temperature of pyrolytic conversion of oil to gas (170 °C)^51,52^. Therefore, paleo-oil pyrolysis should have occurred before the invasion of external epizonogenic hydrothermal fluids. However, the temperature did not reach the peak pyrolytic temperature of liquid oil^52^. The introduction of the natural gas was related primarily to the precipitation of the III-dolomite (dolomite-quartz veins) because the three-phase S-type inclusions and the P-type fluid inclusions were both observed in the III-dolomite and the quartz.

In addition to the dolomite-quartz vein, the II-dolomite was the earliest host mineral in which large quantities of inclusions were captured. Furthermore, the W-type fluid inclusions are heavily distributed in the boundaries between the “cloudy centers” and the “clear rims”. This feature suggests that the fluid inclusions in the II-dolomite are related to the precipitation of the dolomite^21,53^. The homogenization temperatures of the fluid inclusions in the II-dolomite range from 107 to 212 °C. However, these fluid inclusions are too small to obtain ice point temperature data. Therefore, it is impossible to exclude the influence of re-equilibration or to determine the original trapping temperatures^7^. We infer that the precipitation occurred before the introduction of the natural gas, based on the absence of the P-type and the S-type inclusions in the II-dolomite.

### The new genetic mechanism of natural gas

Based on previous studies of P-type inclusions, this study displays two anomalous phenomena. First, the trapping temperatures and pressures of the P-type inclusions display a wide range and can be divided into two groups (Fig. 8). Second, the burial history shows that the maximum burial temperature of well GS6 did not exceed 230 °C (Fig. 9a), whereas the higher group of methane trapping temperatures are much higher than the maximum burial temperature. Thus, the P-type fluid inclusions were not affected only by the burial temperature.

Experiments on in-reservoir petroleum destruction show that the temperature of the pyrolysis of petroleum commonly exceeds 150 °C, and the peak temperature is always 170 °C to 190 °C^52^. If hydrothermal fluid entered the reservoir at a later time, when the liquid oil had already been pyrolyzed, free methane would have been trapped in hydrothermal minerals, and the trapping temperatures and pressures of free methane would have no significance for gas formation. However, mineral paragenesis shows that the quartz precipitated after the II-dolomite. Moreover, the P-type fluid inclusions are seldom found in the II-dolomite but are abundant in the III-dolomite and the quartz (Fig. 5). In addition, the burial history shows that the heat flow increased suddenly at approximately 260 Ma (Fig. 5). Thus, the time of hydrothermal fluid occurrence should not be later than 260 Ma, whereas the formation temperature was below 140 °C, which is sufficient for the pyrolysis of liquid oil^52^. Therefore, the reservoir did not reach the peak temperature of gas generation before the hydrothermal minerals started to precipitate. The burial history shows that the study area experienced rapid burial from 251 to 238 Ma, and the heat flow have just abruptly increased at the same period (Fig. 9a). This sudden change in burial history indicates that regional tectonic movement may have caused the invasion of the hydrothermal fluids.

**Figure 10:**
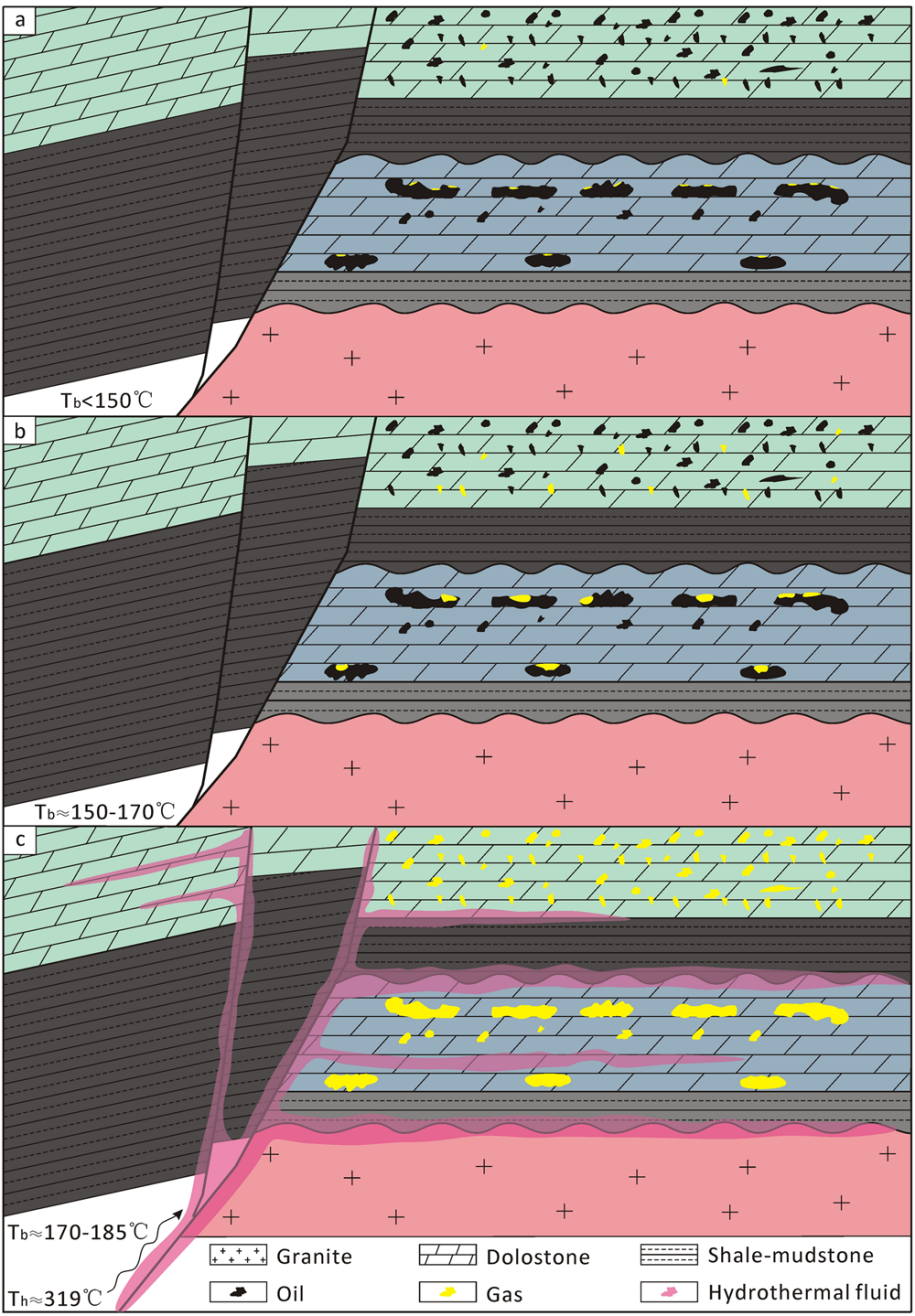
Diagram of the hydrothermal heat mode in the dolostone reservoir. Tb: burial temperature; Th: hydrothermal fluid temperature.

Thus, when the temperature was below 150 °C, little methane became trapped in the reservoir (Fig. 10a). While the reservoir burial temperature was 150–170 °C, little methane was generated by the pyrolysis of petroleum, and the methane was not trapped by the II-dolomite (Fig. 10b). However, with regional tectonic movement (Fig. 9a), hydrothermal fluids (~319 °C) invaded the reservoir (Fig. 10c). The fluid caused the precipitation of hydrothermal minerals, such as the quartz and the III-dolomite, whereas the temperature of the formation water increased^32,54–56^. The high-temperature fluid significantly heated the reservoir and resulted in gas generation.

## Conclusions

Based on the characteristics of diagenetic evolution, mineral paragenesis, and the fluid inclusion, different groups of the W-type fluid inclusions that represent different geological events were recognized and measured. The W-type fluid inclusions in the III-dolomite and the quartz may represent the stages of gas generation. Microthermometry of the P-type fluid inclusions and the coeval W-type fluid inclusions in the quartz were measured. The trapping temperatures of the P-type fluid inclusions show that gas generation was associated with the invasion of hydrothermal fluids, which was induced by regional tectonic movement. Furthermore, mineral paragenesis and the distribution of methane inclusions in the dolomite and the quartz indicate that the hydrothermal fluids invaded before the reservoir reached the peak pyrolysis temperature. Thus, pyrolysis driven by in situ burial generated natural gas, and the invasion of hydrothermal fluids triggered the gas generation process.

## Electronic supplementary material

Dataset 1

## References

[1] Wei G (2013). Formation conditions and exploration prospects of Sinian large gas fields, Sichuan Basin. Petroleum Exploration & Development..

[2] Zou C (2014). Formation, distribution, resource potential, and discovery of Sinian–Cambrian giant gas field, Sichuan Basin, SW China. Petroleum Exploration & Development..

[3] Zhu G, Wang T, Xie Z, Xie B, Liu K (2015). Giant gas discovery in the Precambrian deeply buried reservoirs in the Sichuan Basin, China: Implications for gas exploration in old cratonic basins. Precambrian Research..

[4] Wei G (2014). Characteristics of noble gases in the large Gaoshiti-Moxi gas field in Sichuan Basin, SW China. Petroleum Exploration & Development..

[5] Zhao W, Shen A, Zhou J, Wang X, Junming LU (2014). Types, characteristics, origin and exploration significance of reef-shoal reservoirs: A case study of Tarim Basin, NW China and Sichuan Basin, SW China. Petroleum Exploration & Development..

[6] Li L (2014). The characteristics and implications of late gas accumulation in the Sinian Dengying Formation of Sichuan Basin. Natural Gas Geoscience..

[7] Goldstein RH (2001). Fluid inclusions in sedimentary and diagenetic systems. Lithos.

[8] Pironon J, Bourdet J (2008). Petroleum and aqueous inclusions from deeply buried reservoirs: Experimental simulations and consequences for overpressure estimates. Geochimica Et Cosmochimica Acta..

[9] Aplin AC (1999). Combined use of Confocal Laser Scanning Microscopyand PVT simulation for estimating the composition andphysical properties of petroleum in fluid inclusions. Marine & Petroleum Geology..

[10] Aplin AC (2000). PVTX history of the North Sea’s Judy oilfield. Journal of Geochemical Explorations.

[11] Benchilla L, Guilhaumou N, Mougin P, Jaswal T, Roure F (2003). Reconstruction of palaeo-burial history and pore fluid pressure in foothill areas: a sensitivity test in the Hammam Zriba (Tunisia) and Koh-i-Maran (Pakistan) ore deposits. Geofluids.

[12] Ferket H, Guilhaumou N, Roure F, Swennen R (2011). Insights from fluid inclusions, thermal and PVT modeling for paleo-burial and thermal reconstruction of the Córdoba petroleum system (NE Mexico). Marine & Petroleum Geology..

[13] Gonzalez E (2013). Paleoburial, hydrocarbon generation and migration in the Cordoba Platform and Veracruz Basin: Insights from fluid inclusion studies and 2D modelling. Fire Safety Journal..

[14] Ni ZY (2016). An examination of the fluid inclusions of the well RP3-1 at the Halahatang Sag in Tarim Basin, northwest China: Implications for hydrocarbon charging time and fluid evolution. Journal of Petroleum Science & Engineering.

[15] Jiang H (2014). Tectonic Evolution of the Leshan-Longnvsi Paleo-uplift and Reservoir Formation of Neoproterozoic Sinian Gas. Natural Gas Geoscience..

[16] Xu H (2012). Tectonic evolution of the Leshan-Longnüsi paleo-uplift and its control on gas accumulation in the Sinian strata,Sichuan Basin. Petroleum Exploration & Development..

[17] Song W (1987). Some New Knowledge of Caledonian Paleo-uplift in Sichuan Basin. Atural Gas Industry..

[18] Song H, Luo Z (1995). 1995. The study of the basement and deep geological structures of Sichuan basin, china. Earth Science Frontiers..

[19] Wei G (2015). Tectonic features of Gaoshiti-Moxi paleo-uplift and its controls on the formation of a giant gas field, Sichuan Basin, SW China. Petroleum Exploration & Development..

[20] Chen Z (2017). Biomarker signatures of Sinian bitumens in the Moxi–Gaoshiti Bulge of Sichuan Basin, China: Geological significance for paleo-oil reservoirs. Precambrian Research..

[21] Yang C (2018). Pyrobitumen in South China: Organic petrology, chemical composition and geological significance. International Journal of Coal Geology..

[22] Bodnar, R. J. Revised equation and table for determining the freezing point depression of H_2_O-Nacl solutions. *Geochimica et Cosmochimica Acta* **57**(3), 683–684 (1993).

[23] Warren J (2000). Dolomite: occurrence, evolution and economically important associations. Earth-Science Reviews..

[24] Sibley JM, Gregg DF (1987). Classification of Dolomite Rock Textures. Journal of Sedimentary Research..

[25] Davies GR, Smith LB (2006). Structurally controlled hydrothermal dolomite reservoir facies: An overview. Aapg Bulletin..

[26] Braithwaite CJR, Rizzi G (2010). The geometry and petrogenesis of hydrothermal dolomites at Navan, Ireland. Sedimentology..

[27] Stasiuk LD (1997). The origin of pyrobitumens in upper Devonian Leduc formation gas reservoirs, Alberta, Canada: an optical and EDS study of oil to gas transformation. Marine & Petroleum Geology..

[28] Glikson M, Golding SD, Southgate PN (2006). Thermal evolution of the ore-hosting Isa Superbasin: Central and Northern Lawn Hill Platform. Economic Geology..

[29] Rimmer SM, Crelling JC, Yoksoulian LE (2015). An occurrence of coked bitumen, Raton Formation, Purgatoire River Valley, Colorado, USA. International Journal of Coal Geology..

[30] Cai C, Zhang C, He H, Tang Y (2013). Carbon isotope fractionation during methane-dominated TSR in East Sichuan Basin gasfields, China: A review. Marine & Petroleum Geology..

[31] Hao F (2015). The fate of CO 2 derived from thermochemical sulfate reduction (TSR) and effect of TSR on carbonate porosity and permeability, Sichuan Basin, China. Earth-Science Reviews..

[32] Liu Q (2016). Coupled alteration of hydrothermal fluids and thermal sulfate reduction (TSR) in ancient dolomite reservoirs – An example from Sinian Dengying Formation in Sichuan Basin, southern China. Precambrian Research..

[33] Worden RH, Smalley PC, Oxtoby NH (1995). Gas souring by thermochemical sulfate reduction at 140Â °C. Aapg Bulletin..

[34] Setzmann U, Wagner W (1991). A new equation of state and tables of thermodynamic properties for methane covering the range from the melting line to 625 K at pressures up to 100 MPa. Journal of Physical and Chemical Reference Data..

[35] Zhang C, Duan Z, Zhang Z (2007). Molecular dynamics simulation of the CH 4 and CH 4–H 2 O systems up to 10GPa and 2573K. Geochimica et Cosmochimica Acta..

[36] Lu, H. F. *et al*. Fluid Inclusion. (Science Press). 256–260 (Beijing, 2004).

[37] Zhu C (2009). Quantifying the denudations of major tectonic events in Sichuan basin. Constrained by the paleothermal records. Geology in China..

[38] Richardson N (2008). Extraordinary denudation in the Sichuan basin: Insights from low‐temperature thermochronology adjacent to the eastern margin of the Tibetan plateau. Journal of geophysical research: solid earth..

[39] Wang Y, Jin Z (1999). Progress of the Methods on the Recovery of the Thickness of Eroded Strata in Basin. Advance in Earth Sciences..

[40] Qiu, N., Hu, S., He, L. Principles and Applications on Thermal Regime of Sedimentary Basins. (Petroleum Industry Press). 125–134 (Beijing, 2004).

[41] Wang F, He P, Gao G, Fu J, Liu D (1995). Vitrinite-like macerals existed widespread in high-post mature Cambrian–Ordovician carbonate, and, shale source rocks in China. Journal of the University of Petroleum, China..

[42] Cheng D, Fang J (1997). 1997. Genesis and thermal evolution of vitrinite-like macerals in hydrocarbon source rocks of lower Paleozoic. Petroleum Exploration and Development..

[43] Xiao X, Wilkins R, Liu D, Liu Z, Fu J (2000). Investigation of thermal maturity of lower Palaeozoic hydrocarbon source rocks by means of vitrinite-like maceral reflectance—a Tarim Basin case study. Organic Geochemistry..

[44] Liu S, Sun W, Li Z, Deng B, Liu S (2008). Tectonic uplifting and gas pool formation since Late Cretaceous Epoch, Sichuan Basin. Natural Gas Geoscience..

[45] Deng B (2008). A comparative study of the late Mesozoic uplifting in the eastern margin of Qinghai-Tibet Plateau and Sichuan Basin. China. Journal of Chengdu University of Technology (Science & Technology Edition)..

[46] He B, Xu Y, Wang Y, Luo Z, Wang K (2005). The magnitude of crystal uplift prior to the eruption of the Emeishan basalt: Inferred from sedimentary records. Geotectonica et Metallogenia..

[47] Zhu CQ, Hu SB, Qiu NS, Rao S, Yuan YS (2015). The thermal history of the Sichuan Basin, SW China: Evidence from the deep boreholes. Science China: Earth Sciences..

[48] Chen YJ (2007). Diagnostic fluid inclusions of different types hydrothermal gold deposits. Acta Petrologica Sinica..

[49] Chen YJ, Santosh M, Somreville I, Chen HY (2014). Indosinian tectonics and mineral systems in China: an introduction. Geological Journal..

[50] Cox S, Knackstedt M, Braun J (2001). Principles of structural control on permeability and fluid flow in hydrothermal systems. Reviews in Economic Geology..

[51] Tissot B (1974). Influence of Nature and Diagenesis of Organic Matter in Formation of Petroleum. Aapg Bulletin..

[52] Waples DW (2000). The kinetics of in-reservoir oil destruction and gas formation: constraints from experimental and empirical data, and from thermodynamics. Organic Geochemistry..

[53] Brooks R, Horton AT, Torgesen JL (1986). Occlusion of mother liquor in solution-grown crystals. Journal of Crystal Growth..

[54] White DE (1957). Thermal Waters of Volcanic Origin. Geological Society of America Bulletin..

[55] Groves DI, Goldfarb RJ, Gebre-Mariam M, Hagemann SG, Robert F (1998). Orogenic gold deposits: A proposed classification in the context of their crustal distribution and relationship to other gold deposit types. Ore Geology Reviews..

[56] Kerrich R, Goldfarb R, Groves D, Garwin S (2000). The characteristics, origins, and geodynamic settings of supergiant gold metallogenic provinces. Science China Earth Sciences..

