# Supplementary material for: A new genetic mechanism of natural gas accumulation
Source: Sci Rep. 2018 May 29;8:8336. doi: 10.1038/s41598-018-26517-y (PMC5974082; doi:10.1038/s41598-018-26517-y)
Supplement: Supplementary file 1 — Dataset 1 [file 41598_2018_26517_MOESM1_ESM.docx]

**Title:** A new genetic mechanism for natural gas accumulation

**Authors:** Chengyu Yang^1^, Zhiyong Ni^1,^ *, Tieguan Wang^1^, Zhonghong Chen^2^, Haitao Hong^3^, Long Wen^3^, Bing Luo^3^, Wenzhi Wang^3^

**Institutional affiliations:**

^1.^*State Key Laboratory of Petroleum Resources and Prospecting, China University of Petroleum, Beijing 102249, China*

*^2.^School of Geoscience, China University of Petroleum, Qingdao, Shandong, 266580, China*

^3.^*Exploration and Development Research Institute of Southwest Oil & Gasfield Company, PetroChina, Chengdu, Sichuan 610041, China*

*Corresponding author: Zhiyong Ni

Mailing address: 18 Fuxue Road, Changping District, Beijing 102249, P.R. China

Tel: (+86)-(10)-89739110

Email: nizhy@cup.edu.cn

| Type | Hosting | Size | V_vap._ | T_h_ | Type | Hosting | Size | V_vap._ | T_h_ |
| --- | --- | --- | --- | --- | --- | --- | --- | --- | --- |
|  | Minerals | (μ) | (%) | (℃) |  | Minerals | (μ) | (%) | (℃) |
| W | II-dolomite | 7 | 10 | 123 | W | II-dolomite | 4 | 20 | 149 |
| W | II-dolomite | 7 | 10 | 137 | W | II-dolomite | 5 | 20 | 150 |
| W | II-dolomite | 7 | 10 | 139 | W | II-dolomite | 5 | 20 | 173 |
| W | II-dolomite | 7 | 10 | 161 | W | II-dolomite | 5 | 15 | 189 |
| W | II-dolomite | 7 | 20 | 167 | W | II-dolomite | 6 | 5 | 212 |
| W | II-dolomite | 8 | 20 | 163 | W | II-dolomite | 5 | 10 | 177 |
| W | II-dolomite | 9 | 15 | 159 | W | II-dolomite | 7 | 10 | 189 |
| W | II-dolomite | 7 | 20 | 160 | W | II-dolomite | 7 | 15 | 155 |
| W | II-dolomite | 7 | 15 | 140 | W | II-dolomite | 7 | 10 | 173 |
| W | II-dolomite | 10 | 20 | 141 | W | II-dolomite | 8 | 10 | 149 |
| W | II-dolomite | 10 | 20 | 123 | W | II-dolomite | 7 | 15 | 144 |
| W | II-dolomite | 6 | 10 | 125 | W | II-dolomite | 8 | 15 | 169 |
| W | II-dolomite | 6 | 5 | 145 | W | II-dolomite | 8 | 10 | 172 |
| W | II-dolomite | 5 | 20 | 165 | W | II-dolomite | 9 | 5 | 177 |
| W | II-dolomite | 6 | 10 | 168 | W | II-dolomite | 10 | 5 | 174 |
| W | II-dolomite | 4 | 15 | 197 | W | II-dolomite | 10 | 10 | 167 |
| W | II-dolomite | 4 | 5 | 189 | W | II-dolomite | 7 | 5 | 164 |
| W | II-dolomite | 4 | 15 | 203 | W | II-dolomite | 5 | 5 | 169 |
| W | II-dolomite | 6 | 15 | 136 | W | II-dolomite | 5 | 10 | 173 |
| W | II-dolomite | 4 | 10 | 179 | W | II-dolomite | 5 | 10 | 180 |
| W | II-dolomite | 6 | 20 | 180 | W | II-dolomite | 6 | 15 | 183 |
| W | II-dolomite | 10 | 10 | 163 | W | II-dolomite | 11 | 10 | 169 |
| W | II-dolomite | 7 | 15 | 107 | W | II-dolomite | 7 | 10 | 170 |
| W | II-dolomite | 7 | 10 | 117 | W | II-dolomite | 8 | 10 | 173 |
| W | II-dolomite | 7 | 15 | 118 | W | II-dolomite | 7 | 15 | 176 |
| W | II-dolomite | 6 | 15 | 120 | W | II-dolomite | 9 | 10 | 162 |
| W | II-dolomite | 6 | 10 | 127 | W | II-dolomite | 9 | 10 | 163 |
| W | II-dolomite | 5 | 10 | 133 | W | II-dolomite | 5 | 10 | 168 |
| W | II-dolomite | 6 | 15 | 198 | W | II-dolomite | 5 | 10 | 179 |
| W | II-dolomite | 6 | 10 | 118 | W | II-dolomite | 5 | 15 | 196 |
| W | II-dolomite | 8 | 15 | 115 | W | II-dolomite | 5 | 10 | 170 |
| W | II-dolomite | 6 | 10 | 119 | W | II-dolomite | 10 | 15 | 171 |
| W | II-dolomite | 6 | 10 | 159 | W | II-dolomite | 9 | 10 | 179 |
| W | II-dolomite | 4 | 15 | 175 | W | II-dolomite | 8 | 10 | 129 |
| W | II-dolomite | 8 | 5 | 157 | W | II-dolomite | 8 | 10 | 159 |
| W | II-dolomite | 8 | 10 | 134 | W | II-dolomite | 7 | 10 | 189 |
| W | II-dolomite | 9 | 20 | 119 | W | II-dolomite | 7 | 10 | 145 |
| W | II-dolomite | 7 | 5 | 141 | W | II-dolomite | 6 | 15 | 148 |
| W | II-dolomite | 10 | 5 | 169 | W | II-dolomite | 10 | 10 | 148 |
| W | II-dolomite | 10 | 10 | 150 | W | II-dolomite | 6 | 10 | 149 |
| W | II-dolomite | 9 | 5 | 169 | W | II-dolomite | 6 | 15 | 160 |
| W | II-dolomite | 7 | 10 | 173 | W | II-dolomite | 6 | 10 | 141 |
| W | II-dolomite | 7 | 20 | 155 | W | II-dolomite | 4 | 10 | 165 |
| W | II-dolomite | 10 | 5 | 152 | W | II-dolomite | 6 | 5 | 200 |
| W | II-dolomite | 8 | 10 | 207 | W | II-dolomite | 6 | 10 | 199 |
| W | II-dolomite | 8 | 5 | 200 | W | II-dolomite | 7 | 10 | 198 |
| W | III-dolomite | 6 | 10 | 189 | W | III-dolomite | 10 | 5 | 172 |
| W | III-dolomite | 6 | 5 | 188 | W | III-dolomite | 15 | 5 | 174 |
| W | III-dolomite | 5 | 10 | 189 | W | III-dolomite | 13 | 10 | 201 |
| W | III-dolomite | 5 | 5 | 188 | W | III-dolomite | 5 | 10 | 158 |
| W | III-dolomite | 7 | 15 | 179 | W | III-dolomite | 4 | 10 | 198 |
| W | III-dolomite | 7 | 20 | 204 | W | III-dolomite | 12 | 10 | 223 |
| W | III-dolomite | 7 | 5 | 205 | W | III-dolomite | 11 | 15 | 183 |
| W | III-dolomite | 12 | 10 | 203 | W | III-dolomite | 11 | 15 | 190 |
| W | III-dolomite | 15 | 20 | 159 | W | III-dolomite | 4 | 15 | 193 |
| W | quartz | 13 | 15 | 187 | W | quartz | 12 | 10 | 179 |
| W | quartz | 11 | 5 | 159 | W | quartz | 10 | 15 | 161 |
| W | quartz | 13 | 15 | 158 | W | quartz | 11 | 10 | 173 |
| W | quartz | 12 | 10 | 175 | W | quartz | 11 | 5 | 177 |
| W | quartz | 7 | 10 | 163 | W | quartz | 5 | 10 | 179 |
| W | quartz | 9 | 25 | 165 | W | quartz | 4 | 20 | 189 |
| W | quartz | 6 | 15 | 184 | W | quartz | 8 | 5 | 173 |
| W | quartz | 6 | 5 | 171 | W | quartz | 6 | 10 | 167 |
| W | quartz | 7 | 10 | 168 | W | quartz | 10 | 5 | 169 |
| W | quartz | 6 | 10 | 169 | W | quartz | 10 | 10 | 161 |
| W | quartz | 5 | 20 | 178 |  |  |  |  |  |
